# Targeting NAD Metabolism for the Therapy of Age-Related Neurodegenerative Diseases

**DOI:** 10.1007/s12264-023-01072-3

**Published:** 2023-05-31

**Authors:** Feifei Li, Chou Wu, Gelin Wang

**Affiliations:** 1 School of Pharmaceutical Sciences, Ministry of Education Key Laboratory of Bioorganic Phosphorus Chemistry and Chemical Biology, Tsinghua University, Beijing, 100084 China; 2 Tsinghua-Peking Joint Center for Life Sciences, Tsinghua University, Beijing, 100084 China

**Keywords:** Ageing, Neuroprotection, NAD metabolism, Therapeutic strategy, Neurodegenerative diseases

## Abstract

As the aging population continues to grow rapidly, age-related diseases are becoming an increasing burden on the healthcare system and a major concern for the well-being of elderly individuals. While aging is an inevitable process for all humans, it can be slowed down and age-related diseases can be treated or alleviated. Nicotinamide adenine dinucleotide (NAD) is a critical coenzyme or cofactor that plays a central role in metabolism and is involved in various cellular processes including the maintenance of metabolic homeostasis, post-translational protein modifications, DNA repair, and immune responses. As individuals age, their NAD levels decline, and this decrease has been suggested to be a contributing factor to the development of numerous age-related diseases, such as cancer, diabetes, cardiovascular diseases, and neurodegenerative diseases. In pursuit of healthy aging, researchers have investigated approaches to boost or maintain NAD levels. Here, we provide an overview of NAD metabolism and the role of NAD in age-related diseases and summarize recent progress in the development of strategies that target NAD metabolism for the treatment of age-related diseases, particularly neurodegenerative diseases.

## Brief Introduction to NAD Metabolism

NAD was first discovered in 1906 by Arthur Harden, who found that boiled yeast extract could stimulate fermentation and alcohol production *in vitro* [1]. Over the following decades, NAD was purified, its structure was identified, and its function in hydrogen transfer was revealed. The discovery of NAD was the result of the combined efforts of four Nobel Prize winners [2]. For a long time, NAD was regarded as a cofactor in metabolic pathways. However, in recent years, the discovery of NAD-consuming enzymes such as poly (ADP-ribose) polymerases (PARPs), CD38, sirtuins, and SARM1 (sterile alpha and TIR motif containing 1), has revealed the roles of NAD in other important cellular processes, such as the maintenance of genomic stability, protein modification, epigenetic regulation of gene expression, and immune responses. Consequently, NAD is now regarded as the hub of metabolism, and its critical role in aging and disease development is widely appreciated. Below, we outline the biosynthesis and consumption of NAD, as well as its primary functions in a variety of cellular processes.

### NAD Biosynthesis

**Fig. 1:**
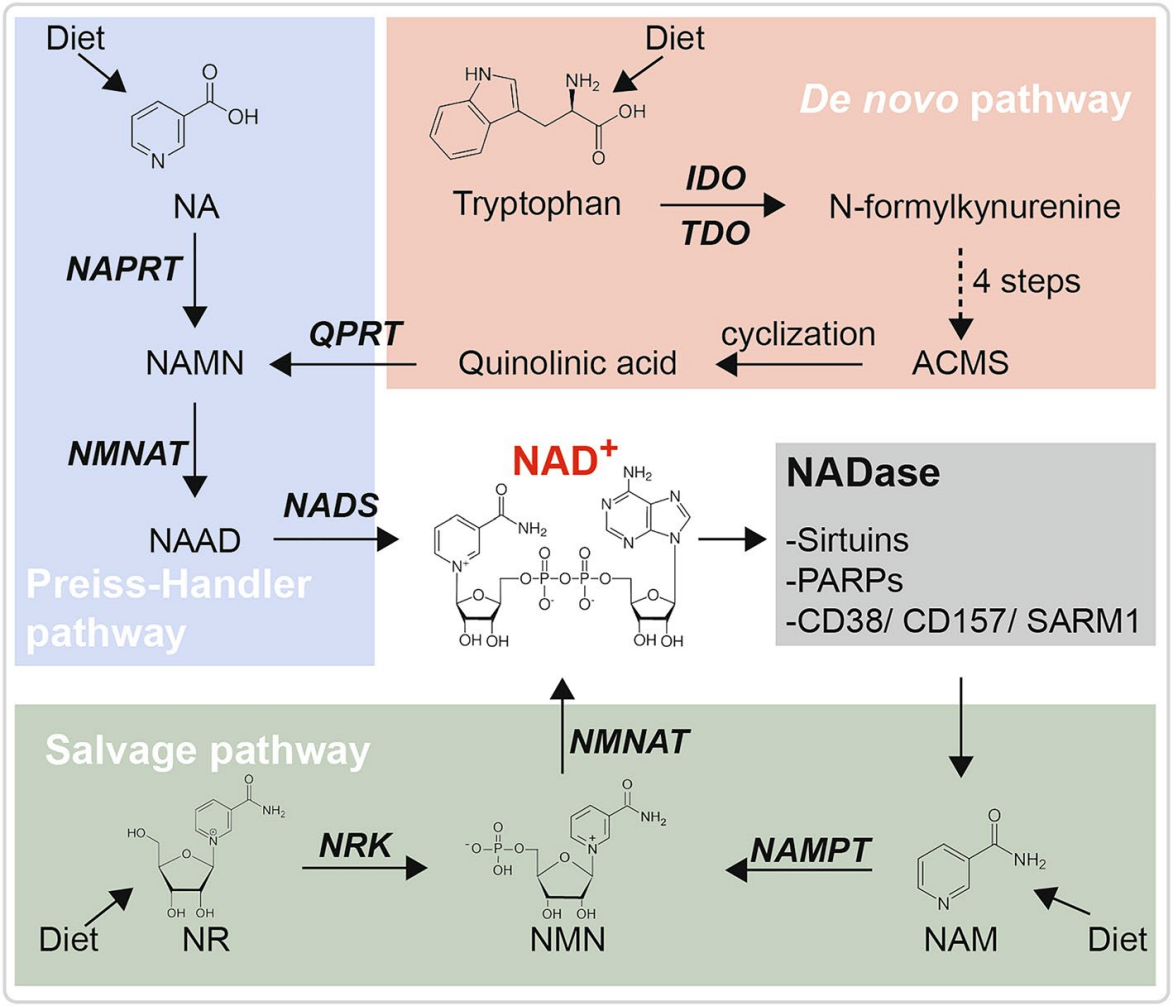
Three distinct pathways for NAD biosynthesis. See the main text for details.

NAD can be synthesized through three distinct pathways (Fig. 1): the *de novo* synthesis pathway (also known as the kynurenine pathway, or KP for short), the Preiss-Handler pathway, and the salvage pathway. The *de novo* synthesis of NAD occurs mainly in the liver and begins with dietary tryptophan. Once taken up into cells by SLC6A19, tryptophan is converted into N-formylkynureine by either indoleamine 2,3-dioxygenase (IDO) or tryptophan 2.3-dioxygenase (TDO). N-formylkynureine then undergoes four enzymatic reactions to form α-amino-β-carboxymuconate-ε-semialdehyde (ACMS). Typically, ACMS is converted to picolinic acid in the presence of ACMS decarboxylase. However, ACMS can spontaneously cyclize to form quinolinic acid (QA), which is then condensed by quinolinate phosphoribosyl transferase into nicotinic acid mononucleotide (NaMN) and enters NAD synthesis through the Preiss-Handler pathway. It is important to note that before being condensed into NaMN, QA is a potent neurotoxin with marked free radical-producing properties [3]. The Preiss-Handler pathway was described in 1958 by Jack Preiss and Philip Handler. In this pathway, three enzymes, namely NA phosphoribosyltransferase (NAPRT), NMN adenylyltransferases (NMNATs), and NAD synthase (NADS), sequentially convert the dietary nicotinic acid into NAD as shown in Fig. 1. The salvage pathway is the primary source of NAD biosynthesis in most mammalian cells. Through the salvage pathway, nicotinamide (NAM), the common product of NAD consumption, is converted back into NAD. NAM is catalyzed to NMN by NAMPT, the rate-limiting enzyme in the salvage pathway. NMN is then converted into NAD by NMNATs. NMN can also be generated by nicotinamide riboside kinase (NRK) from dietary nicotinamide riboside (NR).

### NAD Consumption

NAD plays two fundamental roles in living organisms. Firstly, through the transition between its oxidized and reduced states, NAD/NADH serves as an electron carrier during glycolysis, oxidative phosphorylation, and fermentation, generating ATP to meet the energy demand of the cell, while providing various intermediate metabolites as building blocks for cell growth and proliferation. During the tricarboxylic acid (TCA) cycle, NAD is converted to NADH, along with the generation of various intermediate metabolites. Subsequently, NADH is converted back to NAD during oxidative phosphorylation, establishing an electron gradient across the inner mitochondrial membrane, which is then used for ATP generation by ATP synthase (complex V) [4, 5]. During this process, the overall levels of NAD and NADH do not change, but metabolic stress can affect the NAD/NADH ratio.

**Fig. 2:**
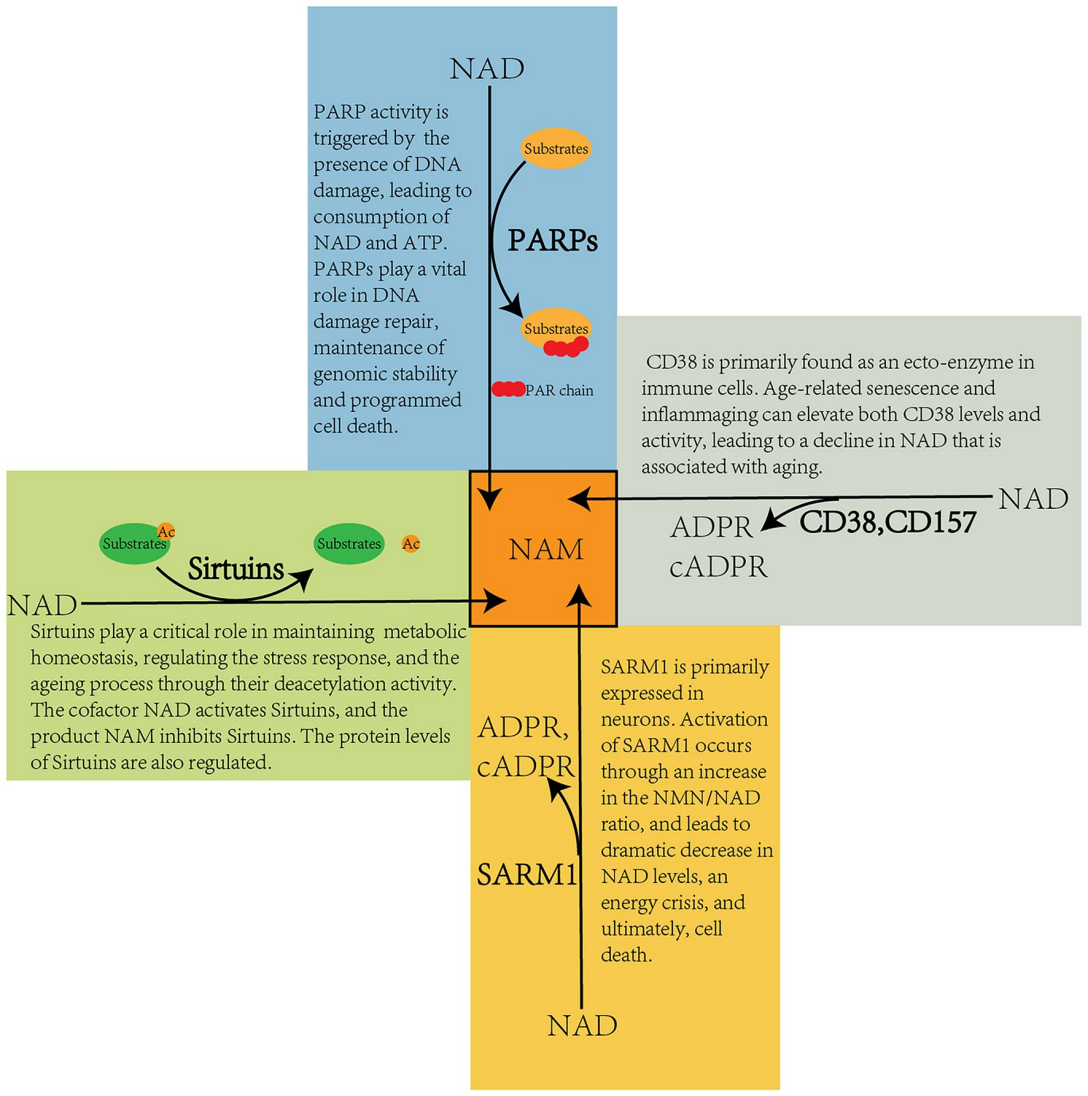
Main NAD-consuming enzymes. Four classes of enzymes mediate the net catabolism of NAD. NAM, the common product of NAD catabolism, is salvaged back to generate NAD as shown in Fig.1.

Secondly, NAD plays a crucial role in cellular processes by participating in pathways regulated by NAD-consuming enzymes, including Sirtuins, PARPs, CD38/157, and SARM1 as shown in Fig. 2. Sirtuins (SIRT1-7) are NAD-dependent deacetylases that modify various target proteins, such as histone, peroxisome proliferator-activated receptor-gamma coactivator (PGC)-1α, p53, NF-kB, FOXOs, and PARP [6]. They regulate a wide range of processes, including transcription, energy metabolism, circadian rhythm, DNA repair, and inflammation [7, 8]. As the sirtuin cofactor, NAD can have a significant impact on the cellular processes mentioned above, and it appears that NAD's positive role in healthy aging is mostly mediated by elevating sirtuin activity. PARPs are another class of NAD consumer that utilizes NAD as a substrate to catalyze the transfer of the ADP-ribose group from NAD to target proteins, a reaction called PARylation or poly ADP-ribosylation. There are 17 PARP isoforms in humans, PARP1 being the most extensively studied and well-known one [9]. PARP1 plays a critical role in DNA repair and cellular stress responses by catalyzing the PARylation of itself and other proteins after DNA damage caused by UV radiation, reactive oxygen species (ROS), environmental factors, or replication errors. These proteins serve as a scaffold to recruit DNA repair proteins to the site of damage, facilitating the initiation of DNA damage repair mechanisms [9]. The PARP1-mediated PARylation modification is a highly ATP- and NAD-consuming process, and excessive PARP activation results in ATP and NAD depletion, leading to cell death termed parthanatos [10]. CD38 and its paralog CD157 are ectoenzymes that catalyze the breakdown of NAD into NAM and ADP-ribose (ADPR) or cyclic ADP-ribose (cADPR). Initially, CD38 was identified as a surface marker of immune cells, but subsequent research revealed its expression in other cell types, such as endothelial cells and neural cells. Studies have suggested that aging-associated CD38 overexpression is the primary cause of NAD decline [11, 12]. SARM1 is a recently-discovered evolutionarily-conserved NAD hydrolase in the neuronal system [13, 14]. Under normal physiological conditions, the NADase activity of SARM1 is self-inhibited. However, insults that elevate the cellular NMN/NAD ratio can relieve the self-inhibitory status of SARM1 and trigger its NADase activity. This activation causes a significant reduction in neuronal NAD levels, which can lead to metabolic catastrophe and ultimately, neuronal degeneration [15]. The consumption of NAD by NAD-consuming enzymes results in a net decrease in NAD levels, NAM being the common by-product of this process. The NAM is then utilized by the NAD salvage synthesis pathway to regenerate NAD and maintain stable cellular NAD levels. Disruptions of NAD homeostasis have been associated with the development of many diseases, particularly age-related diseases.

## The Role of NAD in Aging and Age-Related Neurodegenerative Diseases

**Fig. 3:**
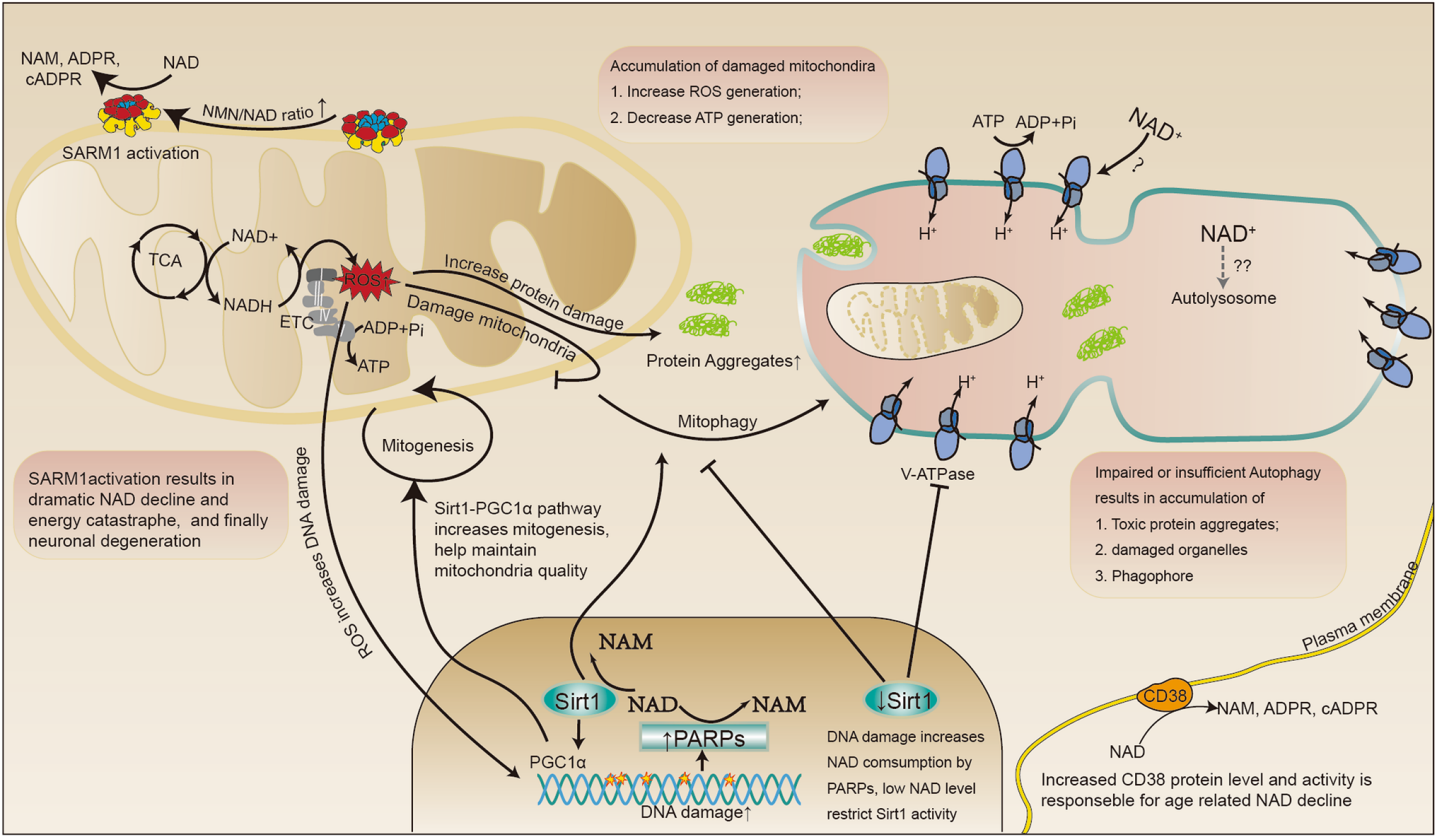
The impact of NAD on the cellular processes involved in aging and age-related neurodegenerative diseases. Oxidative stress, mitochondrial dysfunction, and autophagy impairment are cellular mechanisms shared by neurodegenerative diseases. Accumulated DNA damage activates PARP activity, which increases NAD consumption in the nucleus. The decline in NAD reduces SIRT1 activity, leading to decreased normal mitogenesis and mitophagy. The accumulation of damaged or low-quality mitochondria results in the generation of more mitochondrial ROS, which attacks important macromolecules like lipids, DNA, and proteins, further increasing cellular stress to repair damaged DNA, and clear damaged mitochondria and proteins through autophagy. Impaired autophagy-lysosome pathway activity is frequently reported in neurodegenerative diseases. The direct impact of NAD on the autophagy process needs further investigation, although several cues indicate that the lysosome acidification process may be influenced by NAD.

Aging is characterized by a progressive loss of physiological integrity, leading to increased susceptibility to a range of diseases, including cancer, diabetes, cardiovascular disorders, and neurodegenerative diseases, which are also known as age-related diseases [16]. The hallmarks of aging include genomic instability, telomere attrition, epigenetic alteration, loss of proteostasis, dysregulated nutrient sensing, mitochondrial dysfunction, cellular senescence, stem cell exhaustion, and altered intracellular communication [16]. As the hub of metabolism, NAD plays a crucial role in maintaining the health and well-being of organisms ranging from *Drosophila* to mammals. The decline in NAD levels during aging is widely acknowledged [17]. In this section, we summarize recent progress made in understanding the causes of NAD decline during aging and elucidate how this decline renders organisms susceptible to a variety of age-related diseases, especially neurodegenerative diseases (Fig. 3).

### Mechanisms of NAD Decline during Aging

The decline in net NAD levels can result from either decreased synthesis or increased consumption of NAD. According to an isotope-tracing study, circulating NAD precursor levels are largely unchanged and the *de novo* NAD synthesis from tryptophan is unimpaired during aging. Instead, the primary factor contributing to NAD decline appears to be an accelerated rate of NAD consumption [18]. Consistent with this, another study found that CD38 expression and activity are increased in various tissues, including the liver, adipose tissue, spleen, and skeletal muscles, during aging [19]. CD38-mediated NAD consumption has been suggested to be the main cause of NAD decline during aging. Subsequent studies have revealed that increased inflammation and senescence-associated secretory phenotype (SASP) during aging induce CD38 expression in both endothelial cells and macrophages, leading to increased NAD consumption [11, 20, 21]. Hindered NAMPT expression has also been found in senescent cells, and this further decreases NAD levels by blocking its salvage synthesis [22, 23].

### NAD Dysregulation in the Development of Neurodegenerative Diseases

As the hub of metabolism, NAD dysregulation has a significant impact on energy homeostasis, leading to serious age-related metabolic diseases such as diabetes, cardiovascular diseases, neurodegenerative diseases, and cancer. Here, we focus on the impact of NAD on neurodegenerative disease. As the most sophisticated and energy-intensive system in the body, the nervous system experiences a decline in NAD levels during normal aging [24] and during the progression of neurodegenerative diseases [25]. The decline in NAD levels occurs even before the onset of neurodegenerative symptoms [26], highlighting its contributory effects on disease progression.

The most well-known age-related neurodegenerative diseases include Alzheimer’s disease (AD), Parkinson’s disease (PD), and amyotrophic lateral sclerosis (ALS). The common feature of these neurodegenerative diseases is the loss of axons before neurons die [27, 28]. In the typical injury-induced Wallerian degeneration, the loss of NMNAT2, a critical enzyme in the NAD salvage pathway, results in an increased NMN/NAD ratio. This, in turn, activates the NADase activity of SARM1, leading to NAD depletion and eventual axon degeneration [15, 29]. Activation of SARM1 NADase activity has also been reported to play a role in axon loss during neurodegenerative diseases [30]. The expression of Wlds, a chimeric Ube4b/NMNAT1 fusion protein responsible for the “degeneration slow” phenotype after axon injury, has been reported to protect mice from Parkinson’s and Charcot-Marie-Tooth neuropathy [28]. SARM1 knockout has profound neuroprotective effects in several neurodegeneration models, such as diabetic peripheral neuropathy [31], 6-hydroxydopamine-induced loss of dopaminergic axons in the medial forebrain bundle [32], and a mouse model of retinal ganglion cell degeneration [33]. Inhibitors of SARM1 NADase activity are currently under development for the treatment of neurodegenerative diseases (see the section on NAD-boosting strategies).

Dysregulated mitochondrial homeostasis and function resulting from NAD decline are also common in neurodegenerative diseases [34]. As noted earlier, the nervous system is highly energy-demanding and therefore relies heavily on the normal functioning of mitochondria. NAD plays a crucial role, not only in the oxidative phosphorylation function of mitochondria but also in the regulation of mitochondrial homeostasis by increasing mitochondrial-nuclear communication and activating the mitochondrial unfolded protein response [35, 36]. Maintaining normal NAD/NADH levels is critical for the normal integrity and function of mitochondria. In an age-related glaucoma model, Williams et al. found that mitochondrial dysfunction associated with a decline in NAD is one of the first changes within retinal ganglion cells (RGCs) before the onset of disease characteristics [37]. Phenothiazine has been reported to protect the chronic rotenone model of PD by maintaining normal NAD/NADH levels [38].

The relationship between NAD, mitochondria dysfunction, and neurodegeneration was first revealed in xeroderma pigmentosum group A, ataxia-telangiectasia, and Cockayne syndrome, three DNA-repair disorders with severe neurodegeneration [34]. The accumulation of DNA damage in these diseases causes hyperactivation of PARP1, which dramatically consumes NAD. Restricted NAD availability decreases activation of the NAD-SIRT1-PGC-1α axis, impairs mitophagy, and results in the accumulation of damaged mitochondria. NAD supplementation or inhibition of PARP1 activity rescues the mitochondrial phenotype and extends the lifespan [34, 39]. Consistent with this, NAD supplements stimulate mitophagy and accelerate the clearance of damaged mitochondria, as well as the clearance of aggregated amyloid-β and tau protein in AD models [39–41]. In another study, decreased alpha-synuclein aggregation by NAD-dependent SIRT3 induction was also reported to increase mitochondrial bioenergetics [42].

The role of mitochondrial dysfunction in neurodegeneration is not well understood. One possibility is that damaged mitochondria have a lower bioenergetic rate, which fails to meet the high energy demand of the neurons. Alternatively, damaged mitochondria may result in more oxidative stress in neurons or both. Oxidative stress is widely recognized as a crucial factor in the onset and progression of neurodegenerative diseases [43]. Environmental toxins that introduce oxidative damage, such as rotenone and paraquat, are also risk factors for the development of neurodegenerative diseases. In a mouse model of Leigh syndrome, which is a severe mitochondrial neurodegenerative disease, continuous exposure to low oxygen levels (11% O_2_) prevents neurodegeneration and leads to a dramatic extension of the lifespan. This may indicate the contributory effect of oxidative stress in the development of neurodegeneration [44]. Several antioxidants or radical scavenging agents have shown protective effects in preclinical models of neurodegeneration. In a rotenone-induced PD model, the antioxidant compound idebenone decreases lipid peroxidation and mitigates motor neuron impairment [45]. The free-radical scavenger phenothiazine has also been shown to protect against rotenone-induced neuronal toxicity by reducing protein thiol oxidation [38]. 1-Methyl-4-phenylpyridinium (+)-induced blockade of the electron transport chain and reduction in NAD/NADH levels are reversed by mitochondrial uncoupling and the antioxidant agent embelin, which has a neuronal protective effect in the mouse PD model [46]. NAD/NADH itself has been reported to counter oxidative stress derived from the environment and mitochondria as a redox buffer [47]. In addition, elevating NAD levels can decrease ROS levels by improving mitochondrial quality to decrease ROS generation and increase mitochondrial manganese superoxide dismutase function to accelerate ROS elimination [48]. However, the positive effects of several radical-scavenging agents have not been successfully translated into the clinic, possibly because antioxidants cannot reverse established damage to proteins and organelles [49]. Co-treatment with autophagy-inducing strategies, for instance, autophagosome tethering compounds [50], has been suggested to accelerate the clearance of damaged proteins and organelles [49]. Decreased mitochondrial respiratory capacity has also been reported in AD neurons [51]. Nicholls suggested in 2008 that the deleterious consequence of restricting ATP-generating capacity greatly outweighs that of superoxide radicals in intact neurons [52]. Given the highly polarized structure of neuronal cells, damaged mitochondria and insufficient energy generation pose a significant challenge to the long axon terminals. In summary, NAD decline-induced mitochondrial dysfunction can threaten the healthy status of the nervous system by decreasing energy generation and increasing oxidative stress. Whether other mitochondria-related processes, such as the induction of apoptosis or dysregulation of Ca^2+^ homeostasis, contribute to neurodegeneration requires further study.

Aberrant protein aggregation in the nervous system is another well-known hallmark of neurodegenerative diseases, such as α-synuclein aggregation in PD, amyloid β and Tau aggregation in AD, and TDP (TAR DNA binding protein)-43 aggregation in ALS. For a long time, these aggregated proteins were considered the cause of these neurodegenerative diseases. However, decades of drug development targeting these aggregates have largely failed in the clinic. In recent years, growing evidence suggests that the accumulation of protein aggregates in the nervous system is linked to lysosome dysfunction and the inadequate clearance of misfolded or damaged proteins by autophagy [53]. Neurons are especially vulnerable to protein misfolding and aggregation. Strategies aimed at enhancing the autophagic clearance of aggregated proteins were regarded as the future hope for AD patients. The precise relationship between NAD and autophagy regulation in the nervous system is still largely unknown. Studies conducted in other tissues have shown that sufficient NAD levels are required to maintain lysosomal acidification in heart tissue [54]. A study conducted in breast cancer cells showed that a reduction in SIRT1 levels decreases the expression of the vacuolar-type-ATPase subunit and impairs normal lysosome acidification [55]. In the nervous system, supplementation with the NAD precursor NAM enhances lysosome/autolysosome acidification and reduces autophagosome accumulation in the brains of AD mice [56]. In addition, it has been found that the toxic prion protein induces dramatic NAD depletion, abnormal autophagy activation, and finally neuronal demise [57]. While the exact causal relationship between NAD depletion and abnormal autophagy activation is not yet well established, studies suggest that oxidative stress, which is increased by NAD decline, can accelerate the accumulation of damaged and misfolded proteins, increasing the autophagic stress in the nervous system [49]. Furthermore, the decline in NAD levels has been shown to contribute to the formation of protein aggregation through the nudix homology domain (NHD) that binds NAD. NHD exists in a wide range of proteins and plays a critical role in regulating protein-protein interactions [58]. For example, decreased NAD causes the NAD-binding protein DBC1 to form a complex with PARP1, thus paralyzing its function in DNA damage repair [58]. Aged mice have an increased amount of the DBC1-PARP1 complex, lower PARP1 activity, and increased DNA damage while increasing NAD levels by NMN supplementation can decrease the DBC1-PARP1 complex and reverse all these effects [58, 59]. Thus, a reduction in NAD levels may increase the propensity to form protein complexes through the widely present NHD domain, impairing protein function and increasing autophagic stress at the same time.

Dysregulated cellular processes, including NAD decline, mitochondrial dysfunction, insufficient energy supplementation, redox stress, and decreased autophagy clearance, are the primary drivers of age-related neurodegenerative diseases. These processes can exist prior to the onset of protein aggregates and neurodegenerative symptoms. In general, a decrease in NAD levels disrupts mitochondrial homeostasis, leading to the accumulation of damaged mitochondria. This accumulation can result in inadequate ATP generation and increased ROS generation, which further accelerates NAD decline, mitochondrial damage, DNA damage, and protein aggregation. There is no fixed order of appearance for these events, and all factors are interconnected during the progression of neurodegenerative diseases (as shown in Fig. 3). The appearance of one factor can accelerate the emergence of another, disrupting the homeostatic balance. Maintaining proper NAD levels can increase the ability of the nervous system to counter internal or external disturbance.

## Boosting NAD by Increasing NAD Synthesis

Maintaining normal NAD levels is crucial for an organism’s health and homeostasis. Therefore, strategies aimed at boosting or maintaining normal NAD levels are theoretically beneficial. The therapeutic potential of boosting NAD in aging and age-related diseases has been well appreciated [60]. Numerous strategies for boosting NAD have been explored with the hope of curing or delaying neurodegenerative diseases (Table 1).

**Table 1:** Summary of strategies for boosting NAD biosynthesis in the treatment of aging and age-related diseases.

Strategy/Targets	Compounds	Diseases	Effects	Ref.
**NAD precursor**				
	NAD	6-OHDA-induced PD model	Direct NAD supplement decreased neuronal damage in a mouse PD model	[61]
	NAD	Schinzel-Giedion syndrome (SGS)	The neurodegeneration caused by inheritable DNA damage in SGS neurons can be alleviated by NAD supplement	[62]
	NAD NADPH	Ischemic stroke	The combination of NAD and NADPH provided a greater beneficial effect and a larger therapeutic window in the animal stroke model, by relieving metabolic stress	[63]
	NAM	glaucoma	NAM supplement protected neurons from mitochondrial and metabolic dysfunctions; decreased the likelihood of glaucoma development by ~10-fold, and protected the optic nerve from excavation and axon loss.	[64, 65]
	NAM	Mouse 3xTgAD model	NAM treatment for 8 months improved cognitive performance in AD mice and reduced Aβ and pTau accumulation in AD mouse brains.	[56]
	NAM	*Drosophila* AD model	NAM supplement rescued mitochondrial defects, protected neurons from Aβ toxicity, and reduced behavioral impairments	[66]
	NAM	α-synuclein *Drosophila* PD model	High-dose NAM supplement decreased oxidative stress, increased mitochondrial function, and improved motor function	[67]
	NMN	Retinal detachment	NMN administration had a neuroprotective effect on photoreceptors after retinal detachment and oxidative stress.	[68]
	NMN	Retinal ischemia/reperfusion model	NMN supplement significantly suppressed retinal functional damage and inflammation.	[69]
	NMN	ischemia	NMN dramatically protected the hippocampal CA1 from ischemic injury and showed strong protective effects against ischemic brain injury.	[70]
	NR	3xTgAD/polβ(+/-) mice	NR improved the cognitive function of AD mice and decreased brain DNA damage, neuroinflammation, and apoptosis while increasing SIRT3 activity in AD brains.	[71]
	NR	Tg2576 mouse AD model	NR treatment enhanced PGC-1α expression, β-secretase degradation in the brain, and improved cognitive function and synaptic plasticity.	[72]
	NR	APP/PS1 mouse AD model	NR increased AD brain NAD levels, decreased neuronal inflammation, induced mitophagy, and improved cognitive and synaptic function	[73]
	NR	Mouse aging	NR delayed neuronal stem cell senescence and increased mouse lifespan	[74]
	NR	NMDA-induced brain damage	Intracortical administration of NR reduced brain damage induced by NMDA injection. And delayed NMDA-induced axon degeneration in cultured neurons.	[75]
	NR	Acute/chronic RGC damage	NR enhanced RGC survival in both optic nerves crush-induced acute RGC damage and ocular hypertension-induced chronic RGC damage.	[76]
	NR	ALS	NR supplement delayed motor neuron degeneration and decreased neuroinflammation in hSOD1-linked ALS mouse model.	[77]
	NR	ALS	NR activated the mitochondrial unfolded protein response and improved neurogenesis in adult ALS mouse brains.	[78]
	NRC	ischemia	NRC increased energy supply and promoted cognitive function recovery after mouse brain ischemia	[79]
**NAMPT activators**				
	P7C3-A20	Traumatic brain injury model (TBI)	P7C3-A20 was neuroprotective and promoted endogenous reparative strategies after TBI.	[80]
	P7C3-S243	TBI	P7C3-S243 blocked axon degeneration and preserved normal synaptic activity, learning, and memory in TBI mice	[81]
	P7C3-S243 P7C3-A20	6-OHDA model of PD	Compounds blocked dopaminergic neuron death and preserved normal motor behavior.	[82]
	P7C3	Sciatic nerve crush injury model	P7C3 treatment doubled the neuron survival period after injury and promoted axon regeneration.	[83]
	P7C3-A20	Paclitaxel-induced -peripheral neuropathy	P7C3-A20 treatment provided robust neuroprotection towards paclitaxel-induced peripheral neuropathy	[84]
	P7C3-A20	Ischemic stroke model	P7C3-A20 administration significantly improved stroke-induced damage even when taken 6 hours after ischemia	[85]
	NAT, NAT-5R	Paclitaxel-induced peripheral neuropathy	NAT and NAT-5R alleviated paclitaxel-induced peripheral neuropathy in mice.	[86]
**NADH dehydrogenase modulators**				
	β-lapachone	Age-related hearing loss, Cisplatin-induced hearing loss	β-lapachone improved age-related hearing loss and cisplatin- induced hearing loss by increasing NAD levels through modulation of NQO1 activity	[87, 88]
**Natural products in boosting NAD**				
	Apigenin	LPS-induced neurotoxicity	Apigenin protected mice from LPS-induced neurotoxicity and cognitive decline, which may act by modulating NAD/NADH levels and boosting of SIRT3 activity.	[89, 90]
	Fisetin	6-OHDA-treated SH-SY5Y cells	Fisetin protected neuronal cells from 6-OHDA-induced apoptotic cell death by modulating the PI3K-Akt pathway. Fisetin was reported to be a PARP1 inhibitor and SIRT1 activator	[91–93]
	Embelin	PD	Embelin protected MPP(+)-induced N27 dopaminergic cell apoptosis and showed a protective effect in the MPTP mouse model of PD	[46]

The levels of NAD in an organism are maintained through a balance between its synthesis and consumption. Strategies aimed at boosting NAD levels can be categorized into two classes: increasing its synthesis or blocking its consumption. In this section, we summarize recent progress in strategies aimed at accelerating NAD synthesis. In the next section, we discuss strategies for slowing down NAD consumption. As shown in Fig. 1, dietary tryptophan, nicotinic acid, and NAM are the starting materials for NAD synthesis. Therefore, it is reasonable to consider supplementing these precursors to boost NAD production. In addition, the inadequate function of the NAD salvage pathway is also an important cause of NAD decline [78]. Therefore, manipulating the salvage pathway activity or supplementing NAD precursors has been widely studied as a promising NAD-boosting strategies.

### NAD Precursors as Neuroprotective Agents

Several NAD precursors have been used both *in vitro* and *in vivo* to enhance NAD levels. Here, we discuss the efficacy and safety of these NAD precursors. Direct supplementation of NAD has been found to be effective in reducing neuronal damage by alleviating mitochondrial dysfunction in a mouse model of PD [61]. Moreover, direct supplementation of NAD shows neuroprotective effects in Schinzel-Giedion syndrome, a type of juvenile neurodegenerative disease [62]. In multiple ischemic stroke models, the combination of NAD with a small dose of NADPH has a more potent neuroprotective effect by increasing ATP levels and decreasing ROS levels [63]. However, in an NMDA-induced excitotoxicity-related axon degeneration model, direct supplementation of NAD was found to have a weaker protective effect than NR supplementation [75].

The *de novo* synthesis of NAD from tryptophan is a significant means by which cells produce NAD. As illustrated in Fig. 1, the *de novo* synthesis of NAD is a complex process that involves the participation of many enzymes and the generation of various intermediates. Dysregulation of tryptophan metabolism has been implicated in various neurodegenerative diseases. In biomarker-related studies, patients with neurodegenerative diseases have been found to exhibit an imbalance in tryptophan metabolism [94]. Deletion of TDO has been shown to enhance neurogenesis in the hippocampus and subventricular zone of the mouse brain [95], and inhibition of the KP pathway enzymes, such as IDO and TDO, have also been reported to be neuroprotective [96, 97]. Inhibiting the activity of the KP pathway enzyme TDO has also been reported to extend lifespan [98]. It is worth noting that the majority of the neuroprotective effects resulting from KP pathway manipulations are in contrast to its NAD synthesizing property. As the majority of NAD is produced through the salvage pathway, the relationship between diseases and NAD generation from the kynurenine pathway is not well established [99, 100]. A study on NAD synthetic fluxes suggested that the liver synthesizes NAD from tryptophan, releasing NAM into the circulatory system to be used by other tissues [101]. Further investigation is necessary to determine the impact of the KP pathway on NAD metabolism in the context of aging and disease. Nicotinic acid and NAM, the other two dietary NAD precursors, known as vitamin B3, are well known for their ability to cure severe pellagra. The incidence of pellagra decrease as the quality of life improves. Nonetheless, nicotinic acid has been found to possess other pharmacological activities at higher concentrations, such as treating dyslipidemia and age-related neurological disorders, including AD, PD, and Huntington’s disease [102]. In a recent study, treatment with nicotinic acid for 12 months increased muscle strength and mitochondrial biogenesis in 5 patients with progressive external ophthalmoplegia (PEO), which is a type of mitochondrial myopathy [103]. However, there have been fewer studies on the use of nicotinic acid, possibly due to the lack of efficacy demonstrated in two long-term clinical trials [104, 105] and the undesirable side-effects associated with its use [102, 105]

NAM serves as the starting material for the salvage synthesis of NAD, and its supplementation has been reported to have a protective effect in glaucoma [64, 65]. It can also protect against degeneration and alleviate symptoms in a *Drosophila* model of AD [66], as well as decrease oxidative stress, and improve motor function in a *Drosophila* PD model [67]. In the 3xTgAD mouse model, 8 months of NAM supplementation was shown to enhance cognitive performance in AD mice by reducing toxic protein aggregation in brain tissue [56]

The NAD-elevating potential of NMN and its relevant protective effects on neuronal system diseases have also been extensively studied [48, 68, 106]. The protective effects of NMN supplementation against retinal detachment-induced photoreceptor degeneration [68], ischemia/reperfusion-induced retinal damage [69], and ischemia-induced hippocampal CA1 injury [70] have all been confirmed. Mechanistic studies have revealed that the function of NMN involves the prevention of age-related gene expression, enhancement of mitochondrial oxidative metabolism, maintenance of mitochondrial-nuclear communication, the elevation of SIRT1 protein expression/activity, and the SIRT3-dependent global decrease in mitochondrial protein acetylation [68, 107–110].

The safety of administering oral NMN or NMN derivatives has been evaluated both preclinically and clinically. In a preclinical rodent model, administration of 500 mg/kg/day of NMN for 91 days showed no adverse events, while the highest dose (2000 mg/kg/day) led to reduced body weight and diminished food consumption in rats, effects similar to those seen with the reference item nicotinamide riboside chloride (NRC) dosed at 1740 mg/kg/day [111]. In mice, administration of 300 mg/kg/day NMN for 14 days also showed no acute toxicity [112]. In a clinical trial, a microcrystalline unique polymorph β-NMN formula called MIB-626 was found to be well-tolerated when taken once or twice daily at a dose of 1000 mg for 14 consecutive days by overweight or obese adults [113]. A promising effect of NMN has also been shown in preclinical and clinical models of aging, as NMN administration for 40 days by pre-aging mice (16 months) or human volunteers (45–60 years old) significantly increased telomere length in peripheral blood mononuclear cells [114]. In a long-term study in mice, 12 months of NMN administration not only proved to be safe but also suppressed the age-related increase in body weight, enhanced energy metabolism, showed a positive effect on physical activity, and increased insulin sensitivity when compared to normal chow-fed mice [109].

However, the use of NMN to elevate NAD levels has been questioned. Firstly, there is an ongoing debate about whether NMN is cell permeable [115, 116]. Originally, it was believed that NMN was metabolized to NR extracellularly and then taken up by cells [115]. NR was then catalyzed to NMN inside cells by NRK to enter the NAD salvage synthesis pathways [117]. In 2019, the first NMN transporter Slc12a8 was identified [116], but debate concerning the existence and function of slc12a8 continues [115]. Secondly, concerns about the safety of NMN administration have been raised due to recent findings that an increase in NMN/NAD ratio leads to the activation of SARM1, which is undesirable [15]. Despite these concerns, the known beneficial effects of NMN supplements suggest that there may be unknown mechanisms involved in NMN uptake and metabolism that require future investigation.

NR is another NAD precursor that has been extensively studied. Research has shown that, during normal aging, NR can delay neural stem cell senescence and increase the lifespan of mice [74]. A recent phase I clinical trial on PD patients found that oral NR supplementation is safe and results in mild clinical improvement [118]. In several preclinical studies, NR showed protective effects against various nervous system diseases or damage, including AD [71–73], NMDA-induced brain damage [75], retinal ganglion cell damage [76], and an hSOD1-linked ALS mouse disease [77]. In a mouse brain ischemia model, the NR salt NRC was found to increase the energy supply and promote cognitive function recovery [79]. Studies also suggest that, in most AD models, NR exerts its function by accelerating the clearance of protein aggregation, inducing mitophagy, and improving mitochondrial function in neurons [73, 119]. NR supplementation can also activate the mitochondrial unfolded protein response and improve neurogenesis in adult ALS mouse brains [78].

Despite promising results, concerns have been raised about the stability of NR in the circulatory system, and its use for NAD production is limited by the rate-limiting NRK. To overcome these obstacles, Giroud et al. reported that NRH, the reduced form of NR, is a more potent and faster-acting NAD precursor than NR, and can prevent cisplatin-induced acute kidney injury when taken orally [120]. The *in vivo* effects of NAD or other NAD precursors in aging or age-related diseases have also been summarized elsewhere [121, 122].

### Development of NAMPT Activators

The NAD salvage pathway serves as the primary source of cellular NAD. As previously noted, NAMPT and NMNATs are enzymes involved in this salvage pathway, and activating these two enzymes is expected to boost cellular NAD levels. Researchers at the University of Texas Southwestern Medical Center reported the discovery of the first NAMPT activator, P7C3 [123]. They found that P7C3 and its analog, P7C3-A20, enhance the activity of purified NAMPT *in vitro*, increase NAD levels within cells, protect axons after injury, and have protective effects in mouse models of PD, ALS, traumatic brain injury, paclitaxel-induced peripheral neuropathy, neonatal nerve injury, and developmental delays associated with Down syndrome, a genetic disorder causing intellectual disability [80–85, 123–125]. The neuroprotective effects of P7C3 in rodents have been successfully translated to primates [126], and the P7C3 treatment remains effective even when administered one year after traumatic brain injury [127]. The NAD-boosting activity of P7C3 has also been reported to be beneficial in age-related diabetic heart and skeletal muscle [128, 129]. By enhancing NAD salvage pathway activity, P7C3 and its analogs exert a positive impact on mitochondrial function and the overall metabolism of cells, resulting in the reported benefits.

The success of P7C3 instills confidence in the development of NAMPT activators as neuroprotective strategies. Following P7C3 and its analog, another NAMPT activator called SBI-797812 has been reported [130]. This compound activates NAMPT-mediated NMN production in a concentration-dependent manner in the presence of ATP, with an EC_50_ value of 0.37 ± 0.06 μmol/L. However, in the absence of ATP, SBI-797812 slightly inhibits the NMN synthesis activity of NAMPT. Further biochemical analysis has revealed that SBI-797812 induces a significant shift in the reaction equilibrium towards the NAMPT forward reaction. In addition, SBI-797812 relieves the NAD-mediated NAMPT inhibition, thus allowing for boosting NAD levels. Functional validation has confirmed that SBI-797812 increases cellular NMN and NAD levels in cultured cells. An *in vivo* study found a 1.3-fold increase in NAD in the liver [130]. However, this compound is still undergoing optimization [131, 132], and its impact on the nervous system has not been reported yet.

Our recent study screened 50,000 compounds and identified another NAMPT activator, NAT [86], which binds to NAMPT at a 1:1 ratio with a binding constant of ~500 nmol/L. NAT can significantly increase the catalytic activity (V_max_) of NAMPT for its substrates NAM and phosphoribosyl diphosphate (PRPP). Co-crystal structural analysis has revealed that NAT sits at one end of the active channel of the NAMPT homodimer and slightly affects the binding of NAMPT to its substrates. Structural optimization has generated a more potent NAT analog, NAT-5r [133]. A functional study confirmed that NAT and NAT-5r promote NAD salvage pathway activity and induce metabolic reprogramming by increasing glycolysis, the TCA cycle, and fatty acid oxidation processes inside cells. NAT and NAT-5r have been found to be capable of protecting mice from paclitaxel-induced peripheral neuropathy and promoting the proliferation and self-renewal of primary neural stem cells when cultured *in vitro* [86]. The potential use of NAT and NAT-5r in age-related diseases is under investigation.

In addition to developing small-molecular activators, the expression level of NAMPT can also be regulated, and its activity can be regulated through post-translational modification. For example, the NAD-dependent deacetylase SIRT6 has been reported to upregulate NAMPT activity by directly deacetylating NAMPT [134]. In a kidney ischemia/reperfusion model, downregulation of the NAMPT protein levels induced by reperfusion can be prevented by ERK1/2 inhibition through a small-molecular MEK1/2 inhibitor, trametinib [135]. Furthermore, triterpenes extracted from *Panax notoginseng* leaf are capable of protecting against oxygen-glucose deprivation/re-oxygenation-induced ischemia injury in mice and SH-SY5Y cells by up-regulating NAMPT expression [136].

### Manipulation of NMNAT Activity

NMNAT is the enzyme that catalyzes the second step of the NAD salvage synthesis, and normal levels and function of NMNAT are critical in maintaining NAD levels. Mammals have three NMNAT isoforms that are localized in distinct cellular compartments. Specifically, NMNAT1 is found in the nucleus, NMNAT2 is found in the Golgi and Golgi-derived vesicles, and NMNAT3 is found in the mitochondria. Among them, NMNAT2 has the shortest half-life and plays a critical role in preserving the integrity of neuronal axons [137, 138]. Reduced levels of NMNAT2 protein have been reported in various conditions, including aged oocytes [139], the brain tissue of individuals with AD [140, 141], and heart tissue affected by cardiac hypertrophy [142]. In each of these cases, exogenous overexpression of NMNAT2 has been found to have beneficial effects. Overexpression of NMNAT1, which is the nuclear form of NAD synthase, has a protective effect in hTau mice [143], and gene therapy such as AAV-mediated NMNAT2 overexpression shows promising results against glaucomatous neurodegeneration [144]. Studies involving the overexpression of NMNATs have consistently shown positive results, confirming the beneficial effect of increasing the activity of these enzymes. However, there have been no reports of small molecules capable of enhancing the activity of NMNAT2 or slowing its degradation. Haubrich *et al*. developed a high-throughput screening assay to identify NMNAT modulators and found several NMNAT1 inhibitors from a pool of 912 compounds. Among them, 2,3-dibromo-1,4-naphthoquinone was identified as the most potent inhibitor [145]. Although no activators were identified in this screening, it is important to note that the library of 912 compounds is relatively small, and conducting the same screening scheme on a larger library could potentially yield exciting results.

### Generating NAD through NADH Dehydrogenase

NADH dehydrogenases, including NAD(P)H: Quinone Oxidoreductase 1 (NQO1) and cytochrome b5 reductase 3, catalyze the conversion of NADH to NAD. Mice that overexpress these two enzymes exhibit phenotypes similar to those seen with calorie restriction, including increased lifespan, improved physical performance, and decreased chronic inflammation [146]. The natural products β-lapachone and dunnione promote the conversion of NADH to NAD by increasing NQO1 function [88, 147]. Increasing NAD levels with β-lapachone has been found to effectively prevent age-related and cisplatin-induced hearing loss [87, 88]. Modulating NQO1 activity to increase NAD levels also provides protection against chemotherapy-induced nephrotoxicity, small intestinal damage, cardiac dysfunction, and lung fibrosis [147–150]. Overexpression of another NADH dehydrogenase, the NADH-ubiquinone oxidoreductase (NDI1), has been shown to ameliorate the loss of optical nerve axons and retinal ganglion cells in a mouse model of experimental autoimmune encephalomyelitis, protecting mice from permanent visual loss [151, 152], even though the impact of NDI1 expression on NAD levels was not mentioned.

### Natural Products in Neuroprotection

There is a large body of research focused on the therapeutic potential of natural products for the treatment of neurodegenerative diseases. Although the specific targets of these natural products remain unclear, their reported effects include ameliorating oxidative stress through activation of the Nrf2 system, up-regulating NQO1 and heme oxygenase-1 levels, and increasing NAD/NADH levels. Considering the focus of our paper, we only mention a few relevant studies here. Apigenin, a flavonoid found in nature, has been found to be capable of inhibiting CD38 activity and maintaining NAD/NADH levels [89]. Apigenin has been shown to protect mice from LPS-induced neurotoxicity and cognitive impairment by promoting mitochondrial fusion and mitophagy. Its mechanism of action involves maintaining NAD/NADH levels and boosting SIRT3 activity [90]. Fisetin is another plant flavonoid that has been shown to protect against 6-OHDA-induced neuronal cell death through activation of the PI3K-Akt pathway [91]. The neuroprotective effects of fisetin might also be exerted through the inhibition of PARP1 activity [92] and activation of the SIRT1 activity [93]. Another natural product, embelin, has been found to be capable of increasing NAD/NADH levels and enhancing SIRT1-PGC1α activity by its mitochondrial uncoupling effects. These properties of embelin have been found to be beneficial against MPTP-induced PD [46]. The traditional Chinese herbal prescription Dihuang-Yinzi has long been used to treat neurodegenerative diseases such as AD. Investigation into its mechanism of action revealed that the ability to increase NAD content and enhance energy metabolism may contribute to its neuroprotective activity [153].

## Maintenance of NAD Levels by Modulating NAD-Consuming Enzymes

Reducing unnecessary consumption of NAD represents another promising approach for elevating NAD levels. As noted above, enzymes that consume NAD include sirtuins, PARPs, CD38/157, and SARM1. In the following sections, we discuss strategies targeting each of these enzymes individually (Table 2).

**Table 2:** Strategies by modulating NAD-consuming enzymes.

Targets	Compounds	Disease model	Effects	Ref.
Sirtuin activity modulators				
SIRT1 activator	Resveratrol	Cerebral ischemia (CI)	SIRT1 activation modulates neuronal survival in aged CI mice in an Akt-dependent manner	[154]
SIRT1 activator	Sulfonamide derivatives	6-OHDA-treated neuronal cell	SIRT1 activation by sulfonamide derivatives can protect SH-SY5Y from 6-OHDA-induced cell death	[155]
SIRT1 activator	NeuroHeal	Peripheral nerve axotomy	NeuroHeal can activate the pro-survival autophagy process and protect pups from peripheral nerve axotomy by the concomitant activation of SIRT1 and the PI3K/Akt pathway	[156]
SIRT2 inhibitor	AGK2	Ischemic stroke model	SIRT2 inhibitor AGK2 or sirt2 knockout have neuroprotective effects in the transient middle cerebral artery occlusion mouse model	[157]
SIRT2 inhibitor	Thioamide 53		Thioamide 53 can promote neurite outgrowth of Neuro-2a cells	[158]
PARP inhibitors				
	Olaparib	Schinzel-Giedion syndrome (SGS)	The neurodegeneration caused by inheritable DNA damage can be alleviated by PARP1 inhibition	[62]
	10e	PD	10e protects neurons from α-synuclein pre-formed fiber-mediated neurotoxicity and helps maintain normal NAD levels	[159]
	PJ34	Rat forebrain ischemia	PJ34 almost completely inhibits neuroinflammation and reduces CA1 neuronal death by 84%.	[160]
	PJ34	Rotenone-treated *Drosophila*	PARP1 inhibition by PJ34 reduces dSARM expression, and protects *Drosophila* from rotenone-induced loss of locomotor ability.	[161]
	INO1001	Aortic cross-clamping-induced ischemia/reperfusion	INO1001 markedly protects the spinal cord from aortic occlusion-induced injury.	[162]
	INO1001	Brain traumatic injury	INO1001 significantly reduces microglial activation and increases neuronal survival after TBI.	[163]
	INO1001	R6/2 mutant Huntington’s disease mouse model	INO1001 prolongs R6/2 mutant mouse survival, and reduces severe signs of neurological dysfunction compared to vehicle control	[164]
SARM1 inhibitors				
	Berberine Chloride	Chronic acrylamide-induced axon destruction	Berberine treatment significantly ameliorates axonal degeneration, alleviates pathological changes in the sciatic nerve and spinal cord, and improves neurobehavioral symptoms in acrylamide-treated rats.	[165]
	DSRM-3716	Rotenone-induced axon degeneration	SARM1 inhibition by DSRM-3716 rescues rotenone-treated axons that are fated to degenerate	[166]
	NB-3	Nerve injury model Vincristine-induced neuropathy model	NB-3 is covalently conjugated with SARM1 product ADPR and exerts a potent protective effect against nerve injury	[167]
	EV-99	Vacor and vincristine-induced neuropathy	EV-99 covalently binds to C311 of SARM1, and protects axons from vincristine- and vacor-induced neurite degeneration in cultured dorsal root ganglion neurons	[168]
CD38 inhibitors				
	78c	age	78c can reverse age-related NAD decline, and improve age-related physiological and metabolic parameters	[169]
	78c	age	Increases lifespan and health span of naturally aged mice	[170]
	apigenin	Diabetes, cell senescence, neuroinflammation	Apigenin has protective effects in age-related diabetes, age-related cell senescence, and LPS-induced neuroinflammation and cognitive impairment	[89, 171–173]
	MK-0159	Ischemia/reperfusion	MK-0159 shows strong protection against I/R-induced myocardial damage	[174]
Other enzymes				
Phosphodiesterase (PDE)10A	papaverine	Quinolinic acid-treated primary cortical neuron	Papaverine can increase cellular NAD levels, restore mitochondrial membrane potential, reduce ROS levels, and show neuroprotective activity	[175]
PDE4	Roflumilast	Quinolinic acid-treated primary cortical neuron	Roflumilast can increase intracellular NAD content and protects primary cortical neurons from quinolinic acid-induced toxicity	[176]
PED4	Roflupram	MPP(+)-treated neuronal cells	Roflupram protects dopaminergic neurons from MPP(+)-induced apoptotic cell death *via* CREB/PGC1α pathways	[177]
	Roflupram	Rotenone-treated SH-SY5Y cells	Roflupram can increase the NAD/NADH levels, activate lysosome function, and reduce α-syn levels in rotenone-treated neuronal cells.	[178]

### Modulation of Sirtuin Activity

Sirtuins are a class of protein deacetylases that are conserved through evolution and play a critical role in the regulation of metabolism. There are 7 isoforms of sirtuins in humans, known as SIRT1-7, with different cellular localization and functions [179]. The impact of different sirtuin isoforms on aging and age-related diseases is controversial, and whether inhibiting or activating sirtuin activity depends on the specific isoform involved and requires careful consideration on a disease-by-disease basis [180, 181].

Increasing SIRT1 activity has been shown to be neuroprotective, and strategies to elevate SIRT1 protein levels or boost SIRT1 activity are considered potential interventions for aging and neurodegenerative diseases. Elevating SIRT1 protein levels using various methods, including the small molecule A03, AAV-mediated SIRT1 overexpression, and induced SIRT1 expression, have been shown to have protective effects in an AD mouse model [182], N2a cells transfected with amyloid precursor protein [183], RGC neurons [184], and high-fat diet-induced diabetic neuropathy [185]. In a mouse model of cerebral ischemia, activation of SIRT1 was found to exert a neuroprotective effect by activating the JNK/ERK/MAPK/Akt pathway in aged mice [154]. Small-molecule activators of SIRT1, sulfonamide, and its derivatives, were found to protect against 6-OHDA-induced SH-SY5Y cell death [155]. NeuroHeal, an AI-designed compound to target neurodegenerative diseases, has been shown to activate both the SIRT1 and the PI3K/Akt signaling pathways, which converge on FOXO3 deacetylation and phosphorylation. This activation, in turn, leads to the induction of a pro-survival autophagy process that protects rat pups from peripheral nerve axotomy [156]. Knocking out sirt3 in mice also shows significant neuroprotection against ischemia/reperfusion-induced damage or stroke injury. However, this effect has been ultimately attributed to a compensatory rise in the SIRT1 protein levels, rather than the absence of SIRT3 itself [186].

The impact of SIRT2 on aging and age-related diseases remains unclear and controversial, highlighting the need for further research in this area [187]. SIRT2 activity is required for the maintenance of hematopoietic stem cells, oligodendrocyte fate decision, and neuron remyelination during aging [188–190]. Moreover, it has been reported that SIRT2 in oligodendrocytes can be transported into axons *via* exosomes to help neurons maintain ATP homeostasis in axons [191]. However, SIRT2 activity has also been shown to be neurotoxic [182, 192]. Promising protective effects have been reported in a mouse model of ischemic stroke when either sirt2 is knocked out or its activity is inhibited using the SIRT2-specific inhibitor AGK2 [157]. The reported protective effects may occur through multiple mechanisms, including downregulation of the FOXO3 and MAPK signaling pathways [192], as well as blockade of necrosis, which requires SIRT2-dependent RIP1/3 deacetylation [193]. Thioamide 53, another SIRT2-selective inhibitor, has been found to promote neurite outgrowth in Neuro-2a cells while inhibiting the growth of breast cancer cells [158]. Further evaluation is needed to assess the neuroprotective effect of thioamide 53.

As NAD-dependent protein deacetylases, sirtuins have a wide range of substrates and their functions are quite complex. It is reasonable to expect that sirtuins may have complicated effects under different physiological and pathological conditions. Therefore, the development of highly selective inhibitors or activators for different sirtuin isoforms is required, and their use under different conditions must be carefully considered. Basic research aimed at elucidating the physiological roles of sirtuin proteins will be crucial for the successful development and application of sirtuin modulators in the clinic. In addition, studies on the development of different sirtuin modulators have been reviewed elsewhere [180].

### Modulation of PARP Activity

PARP is another essential NAD consumer, and its overactivation can lead to the depletion of cellular NAD and ATP. Initially, PARP inhibitors were developed for treating BRCA1/2-deficient breast and ovarian cancers due to the synthetic lethality relationship between BRCA1/2 and PARP [194]. Gradually, the potential of PARP inhibitors in treating aging and neuroprotective diseases has also been appreciated. Studies demonstrated that deleting PARP can boost NAD levels, which may have a beneficial impact on AD. Furthermore, in human patients, certain PARP polymorphisms have been associated with reduced risk and severity of AD [66]. PARP inhibition by Olaparib or 10e was found to have neuroprotective effects in a mouse model of transient cerebral ischemia [195] and in *in vitro* neuronal models of PD [159]. Purines, such as hypoxanthine, inosine, and adenosine, can reduce PARP activity [196]. The well-known PARP inhibitor INO1001 has been found to reduce spinal cord injury in an ischemia/reperfusion model [162], protect the brain from traumatic injury [163], and has a neuroprotective effect in the R6/2 mouse model of Huntington’s disease [164]. Another PARP1 inhibitor, PJ34, was reported to suppress neuroinflammation and increase neuronal cell survival in a rat model of forebrain ischemia/reperfusion [160]. PJ34 can also protect *Drosophila* from rotenone-induced locomotor disability by maintaining NAD levels and inhibiting dSARM expression [161].

### Modulation of SARM1 Activity

SARM1 has been identified as a critical mediator of axon degeneration in the nervous system following traumatic injury, as well as in various neurodegenerative diseases. Developing SARM1 inhibitors was assumed to protect the neuronal system from degeneration and was widely viewed as a promising neuroprotective strategy [197]. Deletion of the sarm1 gene in mice was shown to protect against several types of axon degeneration, including diabetic neuropathy [31], traumatic brain injury [198], and vincristine-induced neuropathy [199], giving confidence in the development of small-molecular SARM1 inhibitors. High-throughput screening identified berberine chloride and zinc chloride as the first noncompetitive SARM1 inhibitors with modest potency [200]. The neuroprotective potential of berberine chloride against chronic acrylamide-induced axon destruction was confirmed in a recent study [165]. Subsequently, a more potent and selective isoquinoline inhibitor, DSRM-3716, was discovered and found able to replicate the sarm1 knockout phenotype and protect axons from axotomy-induced or mitochondrial dysfunction-induced degeneration [166]. Additional investigation into the mechanism of action of DSRM-3716 (designated as 1) revealed that a base exchange reaction happens between 1 and the NAM moiety of NAD, leading to the formation of a new compound, 1AD, which is a *bona fide* SARM1 orthostatic inhibitor [197]. Cryo-EM structural studies revealed that the allosteric activator NMN binds to the ARM domain, leading to a significant reorientation of the ARM domain and the formation of a two-stranded TIR domain, as well as an orthosteric site. 1AD binds to the orthosteric site spanning two TIR domain molecules and directly blocks the NADase activity [197]. Bratkowski and colleagues described a new class of SARM1 inhibitors that can intercept NAD hydrolysis by covalently conjugating with ADPR to form small-molecule ADPR adducts, which can effectively inhibit SARM1 activity. The neuroprotective effect of one such inhibitor, NB-3, has been demonstrated in preclinical nerve injury models and a vincristine-induced neuropathy model [167]. Considering that SARM1 is activated by a high NMN/NAD ratio, Sasaki *et al*. proposed that the combination of the NAMPT inhibitor FK866 with nicotinic acid riboside can be used to decrease the NMN level and increase the NAD precursor NaMN level, thereby helping to maintain a low NMN/NAD ratio and effectively blocking SARM1 activation. NaMN has also been reported to be capable of binding to the allosteric pocket of SARM1 and helping maintain the auto-inhibited configuration of the SARM1 ARM domain [201]. The authors suggested that the allosteric binding pocket of SARM1 enables the development of potent SARM1 inhibitors for the treatment of neurodegenerative disorders. Another study by Feldman reported that the tryptoline acrylamide EV-99 can specifically and covalently bind to C311 of the ARM domain, and protect the axon from vacor- or vincristine-induced axon degeneration [168]. Since the NMN/NAD ratio mediates the self-inhibition status of SARM1 [13], agents that can increase NAD levels may have the potential to inhibit SARM1 activity. For instance, Sarkar recently reported that the PARP inhibitor PJ34 can inhibit rotenone-induced SARM1 activation [161].

### Modulation of CD38 Activity

CD38 is an important cell surface ectoenzyme widely expressed in various types of cells and functions as another NAD hydrolase [202, 203]. Reports have shown that age-related upregulation of CD38 is the main cause of NAD decline during aging [19]. Therefore, developing CD38 inhibitors could be a useful strategy for maintaining NAD levels in the aging process. CD38 knockout has been found to decrease AD pathology in an APP/PS mouse model [204] and protects mice against ischemic brain damage [205]. However, CD38 knockout does not affect ALS mouse survival in a hSOD1-linked ALS mouse model [77], indicating that CD38-mediated NAD consumption may not be involved in hSOD1-induced ALS. Tarrago *et al*. reported a potent and specific CD38 inhibitor, 78c, which can reverse the age-related NAD decline and improve age-related physiological and metabolic parameters [169]. Moreover, 78c has been found to be capable of increasing the lifespan and health span of naturally aged mice, improving exercise performance, endurance, and metabolic function [170]. Another study suggested that 78c exerts its activity by forming an adduct with the CD38 product ADPR, and the 78c-ADPR adduct formed acts as a potent CD38 inhibitor [167]. Apigenin, a flavonoid natural product, is capable of inhibiting CD38 activity [89], and its ability to maintain NAD/NADH levels has been confirmed in several studies [89, 171, 172]. The protective role of apigenin has mainly been reported in age-related metabolic disorders such as diabetes [89, 172], age-related cell senescence [173], LPS-induced neuroinflammation, neurotoxicity, and cognitive impairment [90, 171]. Recently, another potent CD38 inhibitor, MK-0159, has been reported. Its IC_50_ against CD38 enzymatic activity *in vitro* is 3 nmol/L [174]. MK-0159 has been found to exert stronger protection against I/R injury-induced myocardial damage than NR or 78c, making it an attractive candidate for various CD38 overexpression-related diseases and conditions.

### Other Enzyme Inhibitors

PAP has been shown to increase cellular NAD levels and exert neuronal protective activity by inhibiting phosphodiesterase-10A [175]. Similarly, the phosphodiesterase-4 (PDE4) inhibitor, Roflumilast, has been reported to increase the intracellular NAD content and has a protective effect against quinolinic acid-induced human primary cortical neuron toxicity [176]. A Roflumilast analog, Roflupram, has also been shown to exert a neuroprotective effect by increasing the cellular NAD/NADH levels and activating lysosomal function [178]. It has been suggested that the neuroprotective effect of PDE4 inhibitors may be attributed to the activation of the CREB/PGC1α pathway and the resulting improvement of metabolic parameters [177]. Nicotinamide N-methyltransferase (NNMT) is an enzyme associated with an impaired NAD salvage pathway and has been found in aged muscles. Inhibitors of NNMT have been reported to increase myoblast regeneration and functional recovery after skeletal muscle injury [206].

## Conclusions

As our understanding of aging expands, the pursuit of healthy aging has become an increasingly desirable goal. However, despite this aspiration, healthy aging remains out of reach for most people. To address this issue, it is crucial that we continue to make new scientific discoveries in order to gain a systematic understanding of the aging process and develop innovative strategies to prevent, delay, or even cure age-related diseases. Furthermore, it is imperative that we effectively translate this research into clinical practice in order to benefit human beings. It is important to note that aging is a systematic and progressive process, and developing one age-related disease can increase the risk of developing additional age-related conditions. As a result, older individuals often suffer from multiple age-related diseases concurrently. Unfortunately, the current medical care system typically treats patients in a disease-specific manner, which can be inefficient. To address this issue, we propose a comprehensive approach to treating age-related diseases, with a focus on boosting NAD levels as an ideal choice.

The decline in NAD levels during aging and age-related diseases has been identified as a targetable process for promoting healthy aging and/or curing or delaying the progression of age-related diseases. Several strategies for boosting NAD levels have been proposed, and we summarized recent advances above. Currently, there are three categories of strategies under development for increasing NAD levels: providing NAD precursors, enhancing NAD synthesis pathway activity, and inhibiting NAD-consuming enzyme activity. It is currently unclear whether any of these strategies is superior to the others. While NAD precursor supplements are the most frequently studied strategy, it is important to note that this approach may not be effective or even harmful if NAD synthesis pathways are blocked in certain diseases. For example, NMNAT2, which is critical for NAD salvage synthesis, has been reported to be downregulated in several neurodegenerative diseases. Therefore, manipulating the activity or protein levels of NAD synthesis enzymes is a second crucial strategy for maintaining a healthy dynamic status of NAD. However, one potential concern with this strategy is the variability in the half-life of NAD across different tissues, which can range from 15 minutes to 15 hours [101]. As the physiological importance of this difference is still unknown, it is important to consider the tissue selectivity of this approach in case the disruption of this difference may result in potential adverse effects. Modulating the activity of NAD-consuming enzymes is the third avenue for increasing NAD levels. Small molecules or antibodies targeting CD38 provide promising activity, as increased CD38 activity has been found to be responsible for the global decline in NAD levels during aging. SARM1 inhibitors are under extensive development now, as activation of SARM1 results in axon degeneration. However, modulators of sirtuins and PARPs need to be developed with caution, as the functions of these two NAD consumers are complicated and sometimes controversial. Many beneficial effects of NAD are exerted by sirtuins, and normal PARP activity is critical in maintaining genomic stability. Therefore, the physiological and pathological functions of different NAD consumers should be carefully considered during the development of their modulators.

Boosting NAD as a potential therapy for age-related neurodegenerative diseases is still in an early stage of development. A better understanding of NAD metabolism and regulation is necessary and requires further basic research. Investigating the roles of various NAD-consuming enzymes in NAD degradation across different tissues and subcellular compartments is crucial. While promising preclinical results exist, there is currently limited evidence of therapeutic benefits in clinical populations. Therefore, it is crucial to continue efforts to successfully translate these findings into clinical practice. In addition, it is still unknown whether one NAD precursor is superior to another, and systematic comparisons are needed. It is also essential to determine whether the use of different NAD precursors should be tailored to specific diseases and stages of the disease.

When developing NAD-boosting strategies, three key points must always be kept in mind. Firstly, since aging and age-related diseases are chronic and require long-term interventions, safety is a vital concern when developing compounds or strategies. A careful examination of the tradeoff between benefits and risk factors is necessary. Secondly, determining a safety range for NAD boosting is essential since excessive levels of NAD can be harmful, as shown by reported oxidative cytotoxicity induced by the potent NAD precursor NRH [207]. Lastly, since NAD is in a dynamic equilibrium between synthesis and consumption, it is perhaps more important to keep NAD in a healthy salvaging state rather than elevating the stable state of NAD levels. With these considerations in mind, safe and efficient NAD-boosting strategies can pave the way for a future of healthy aging.
